# Hemodynamic evaluation of intracranial arteriovenous malformations: Pre- and post-treatment 2D phase-contrast MRI measurements

**DOI:** 10.1177/20584601241269608

**Published:** 2024-08-08

**Authors:** Maria Correia de Verdier, Elisabeth Ronne-Engström, Ljubisa Borota, Johan Wikström

**Affiliations:** 1 Department of Surgical Sciences, Section of Neuroradiology, 59592 Uppsala University, Uppsala, Sweden; 2 Department of Neuroscience, Section of Neurosurgery, 59592 Uppsala University, Uppsala, Sweden

**Keywords:** arteriovenous malformation, brain, MR-imaging, hemodynamics/flow dynamics

## Abstract

**Background:**

Hemodynamic changes are seen in the feeding arteries of arteriovenous malformations (AVMs). Phase-contrast MRI (PC-MRI) enables the acquisition of hemodynamic information from blood vessels. There is insufficient knowledge on which flow or velocity parameter best discriminates AVMs from healthy subjects.

**Purpose:**

To evaluate PC-MRI-measured flow and velocity in feeding arteries of AVMs before and, when possible, also after treatment and to compare these measurements to corresponding measurements in healthy controls.

**Materials and Methods:**

Highest flow (HF), lowest flow (LF), mean flow (MF), peak systolic velocity (PSV), end-diastolic velocity (EDV), and mean velocity (MV) were measured in feeding arteries in patients with intracranial AVMs using 2D PC-MRI at 3 T. Measurements were compared to previously reported values in healthy individuals. Values in patients above the 95th percentile in the healthy cohort were categorized as pathological. Nidus volume was measured using 3D time-of-flight MR angiography.

**Results:**

Ten patients with diagnosed AVMs were examined with PC-MRI. Among these, three patients also underwent follow-up PC-MRI after treatment. Pathological velocities (PSV, EDV, and MV) were seen in all five subjects with a nidus larger or equal to 5.7 cm^3^, whereas pathological flow values were not seen in all, that is, pathologic HF in three, pathologic LF in two, and pathologic MF in two. After treatment, there was a decrease in flow and velocity (all measured parameters). After treatment, velocities (PSV, EDV, and MV) were no longer abnormal compared to healthy controls.

**Conclusion:**

Patients with a large AVM nidus show pathological velocities, but less consistent flow increases. Following treatment, velocities normalize.

## Introduction

Intracranial arteriovenous malformations (AVMs) are believed to be a developmental anomaly of the vascular system. They are characterized by an abnormal tangle of dilated vessels (i.e., a nidus) located between one or more feeding arteries and one or more draining veins. This results in the lack of a normal capillary bed and arteriovenous shunting with a higher flow and velocity than in normal vessels. Although there has been extensive research on novel techniques for quantitative assessment of various AVM hemodynamic parameters, standardized definitions for these parameters and a clear correlation with hemorrhage risk are still lacking.^1^

Today, two imaging modalities are most commonly used in clinical practice to evaluate hemodynamics in patients with AVMs: digital subtraction angiography (DSA) and transcranial Doppler (TCD) ultrasound. In patients with AVMs, the blood flow profile will be disturbed because the decreased resistance leads to increased flow and a change in the flow curve shape. Blood flow can be measured non-invasively with Doppler ultrasound or MRI. There are studies investigating these changes with intraoperative or transcranial Doppler ultrasound.^2,3^ MRI has the possible advantage over Doppler ultrasound of being dependent on neither the user nor the vessel location.

MRI enables the acquisition of hemodynamic information through measurement of blood flow with the phase-contrast MRI technique (PC-MRI).^4,5^ Using this technique, the flow and velocity profiles over the heart cycle can be measured. PC-MRI measurements are used in clinical practice to quantify blood flow and velocities within the heart and great vessels, but pulsatile hemodynamic information from intracranial arteries can also be obtained.^6–8^ There are a few previous studies showing increased mean flow, mean velocity, and peak velocity in feeding arteries compared to the contralateral side and normalization after treatment.^9–14^ The PC-MRI technique has thus the potential to be a non-invasive alternative to DSA for therapy monitoring after AVM treatment. With PC-MRI, it is possible to obtain pulsatile hemodynamic information that yields not only mean values but also shows periodic fluctuations over the cardiac cycle with peak systolic velocity (PSV) and end-diastolic velocity (EDV). It is not known which of these PC-MRI parameters is the most sensitive to the pathological changes seen in feeding arteries in AVMs. To our knowledge, no prior study has investigated which of these PC-MRI parameters to use and compared results in patients with those obtained in large cohorts of healthy subjects.

The aim of this study was to evaluate periodic fluctuations of flow and velocity over the cardiac cycle in feeding arteries of AVMs before and, when possible, also after treatment using PC-MRI. Furthermore, we aimed to compare these measurements to corresponding measurements in healthy controls, and to identify which parameter has the best potential to discriminate normal from pathological.

## Material and methods

### Subjects

Subjects eligible for inclusion were patients with intracranial AVMs diagnosed between 2014 and 2021 and that had a remaining AVM nidus. Patients were examined when included in the study, and repeated examination was performed if the patient received additional treatment for the AVM. Institutional Review Board approval was obtained, and guidelines on patient consent have been met.

### Magnetic resonance imaging

A 3 T scanner (Achieva 3 T X, Philips Medical Systems, Best, the Netherlands) and a 32-channel head coil were used for all the PC-MRI measurements. The intracranial artery that mainly supplied the nidus (feeding artery) was localized with a 3D time-of-flight MR angiography sequence. The feeding artery was defined as the main intracranial arterial branch supplying the nidus (anterior, middle, or posterior cerebral artery; superior cerebellar artery; basilar artery; and anterior or posterior inferior cerebellar artery). Blood flow and velocity information was obtained using a PC-MRI sequence. The measuring plane was placed perpendicular to the feeding artery (middle cerebral artery [MCA] in the M1 segment, posterior cerebral artery [PCA] in the P2 segment, and basilar artery between the origin of the anterior inferior cerebellar artery and superior cerebellar artery and posterior inferior cerebellar artery [PICA] 0.5–2 cm from its origin from the vertebral artery). In arteries where we did not have prior knowledge of the normal ranges of PC-MRI measurements, measurements were also made in the contralateral artery. Repeated measurement with adjustment of placement of the measuring plane was made if there were tortuous vessels or branches causing an inverted intraluminal signal that was not caused by aliasing in the phase difference image.

A gradient-echo T1-weighted PC-MRI sequence was performed, and the following imaging parameters were used: TR/TE 11/6.6 ms, flip angle 15°, bandwidth 55.6 kHz, acquired voxel size 0.59/0.84/4.00 mm, and number of signal averages (NSA) 2. Velocity encoding sensitivity (VENC) was set to 150 cm/s. For improved signal-to-noise ratio, the VENC was reduced and the measurement repeated if the measured peak velocity was below one-third of the preset value. The VENC was increased if there was aliasing caused by phase wrapping. Retrospective gating was used to assess blood velocity at 12 (one subject) or 25 phases (remaining subjects) during the cardiac cycle. Pulse registration was performed with a peripheral pulse unit placed on the subject’s finger. The PC-MRI sequence took approximately 3:45 min but could vary depending on the subjects’ pulse and need for repeated measurement.

### Evaluation

Nidus volume was evaluated using a 3D time-of-flight MR angiography sequence and commercially available software (Vue PACS, version 12.2.8.100.0269, Philips Medical Systems, Best, the Netherlands). PC-MRI data were analyzed using commercially available software (Extended MR WorkSpace 2.6.3.5, Philips Medical Systems, Best, the Netherlands). An elliptic region of interest (ROI) was drawn around the vessel in the phase difference image. ROI size was chosen based on signal properties on an anatomical magnitude image and phase difference image. When necessary for excluding an inverted intraluminal signal, a Bézier ROI with smooth contour line between outlining points was drawn. In this context, an inverted intraluminal signal not caused by aliasing refers to voxels with inverted signal intensity in the phase difference image compared to the surrounding voxels. The outlining points were then adjusted to exclude a visible inverted signal that affected the maximum velocity curve. Flow and maximum velocity were calculated for each of the cardiac phases, with flow defined as mean flow over the ROI and maximum velocity defined as the highest pixel value over the ROI. The results were ordered with the lowest measured value first. Ordered time series of flow and velocity values were used in order to create graphs. Mean flow over the ROI, PSV (defined as the highest maximum velocity), and EDV (defined as the lowest maximum velocity) were calculated for each vessel. Resistivity index (RI) and pulsatility index (PI) were calculated using equations developed by Gosling and Pourcelot, as follows^15,16^:

$$Resistivity\;index=\frac{Peak\;systolic\;velocity-end‐diastolic\;velocity}{Peak\;systolic\;velocity}$$

$$Pulsatility\;index=\frac{Peak\;systolic\;velocity-end‐diastolic\;velocity}{Mean\;velocity}$$

### Statistical analysis

To test for difference between nidus volume and PC-MRI parameters, Spearman rank test was calculated using IBM SPSS Statistics 28 (IBM SPSS, Inc.). The significance level of observed differences was set at *p* < .05. To test for differences between patients and healthy controls, data from our previously published study were used. In this study, PC-MRI measurements were made in the anterior, middle, and posterior cerebral arteries in 30 healthy volunteers with the same imaging and measurement method (except for lower temporal resolution [12 cardiac phases instead of 25] in the healthy controls and the initial three MRI scans in this study [all in the same patient]).^7^ A Gaussian mixture distribution was fitted to each group of measurements from the control group to allow flexible modeling of non-normal data. How extreme the patients with AVMs were was then assessed by checking which quantiles of the fitted distributions their measurements corresponded to. The analysis was carried out using the mixtools package version 1.2.0 in R version 4.1.2.^17,18^

## Results

Twelve patients with diagnosed intracranial AVMs were recruited to the study. Two patients were excluded, one patient did not complete the MRI and one patient did not have a residual nidus visible on MRI, leaving a total of 10 patients included in the study. Patient demographics, AVM characteristics, and treatment parameters are described in Table 1. We obtained usable PC-MRI information in all 10 patients. Three patients underwent treatment and follow-up MRI. A total of 18 MRI examinations are included in the study. In six PC-MRIs (in six different patients) ROI adjustments were made to exclude intraluminal artefacts. In two patients the curve profile was still uneven despite ROI adjustments.

**Table 1.** Patient demographics, AVM characteristics, and treatment parameters.

Age at first MRI	22–68 years (mean 43, median 43)
Symptoms at diagnosis	
- hemorrhage	4 (40%)
- seizures or other neurological symptoms	6 (60%)
Prior treatment of AVM	5^a^ (50%)
- surgery	1 (10%)
- radiation	2 (20%)
- embolization	3 (30%)
Nidus volume at first MRI	0.5–92.9 cm^3^ (mean 15.4, median 4.1)
Main feeding artery	
- middle cerebral artery	5 (50%)
- posterior cerebral artery	3 (30%)
- basilar artery	1 (10%)
- posterior inferior cerebral artery	1 (10%)

^a^One patient was treated with both embolization and radiation.

**Figure 1.**
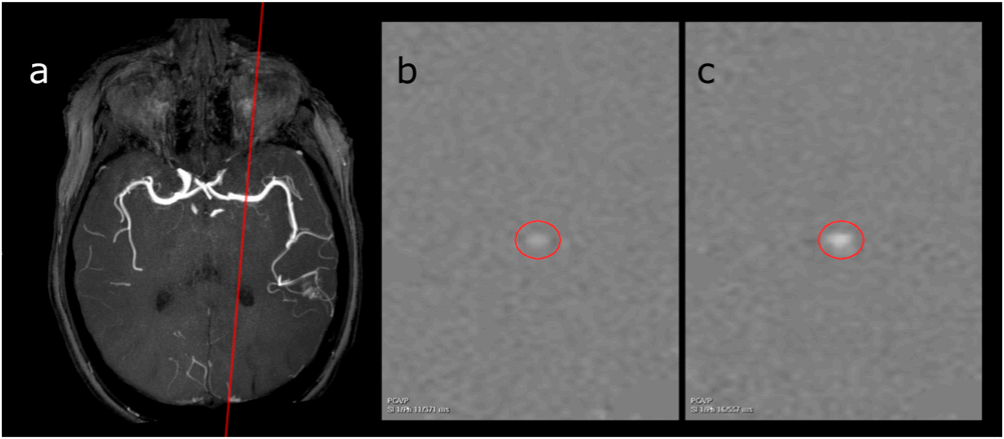
a-c. Subject with an AVM with the middle cerebral artery as feeding vessel. 1 a—Maximum intensity projection of a 3D time-of-flight MR angiography sequence showing the AVM nidus in left parietal lobe. The red line illustrates the placement of the measuring plane. 1 b–c—Phase-contrast MRI (phase difference images) obtained perpendicular to the middle cerebral artery in diastole (1b) and systole (1c). A region of interest (ROI) is surrounding the middle cerebral artery.

**Figure 2.**
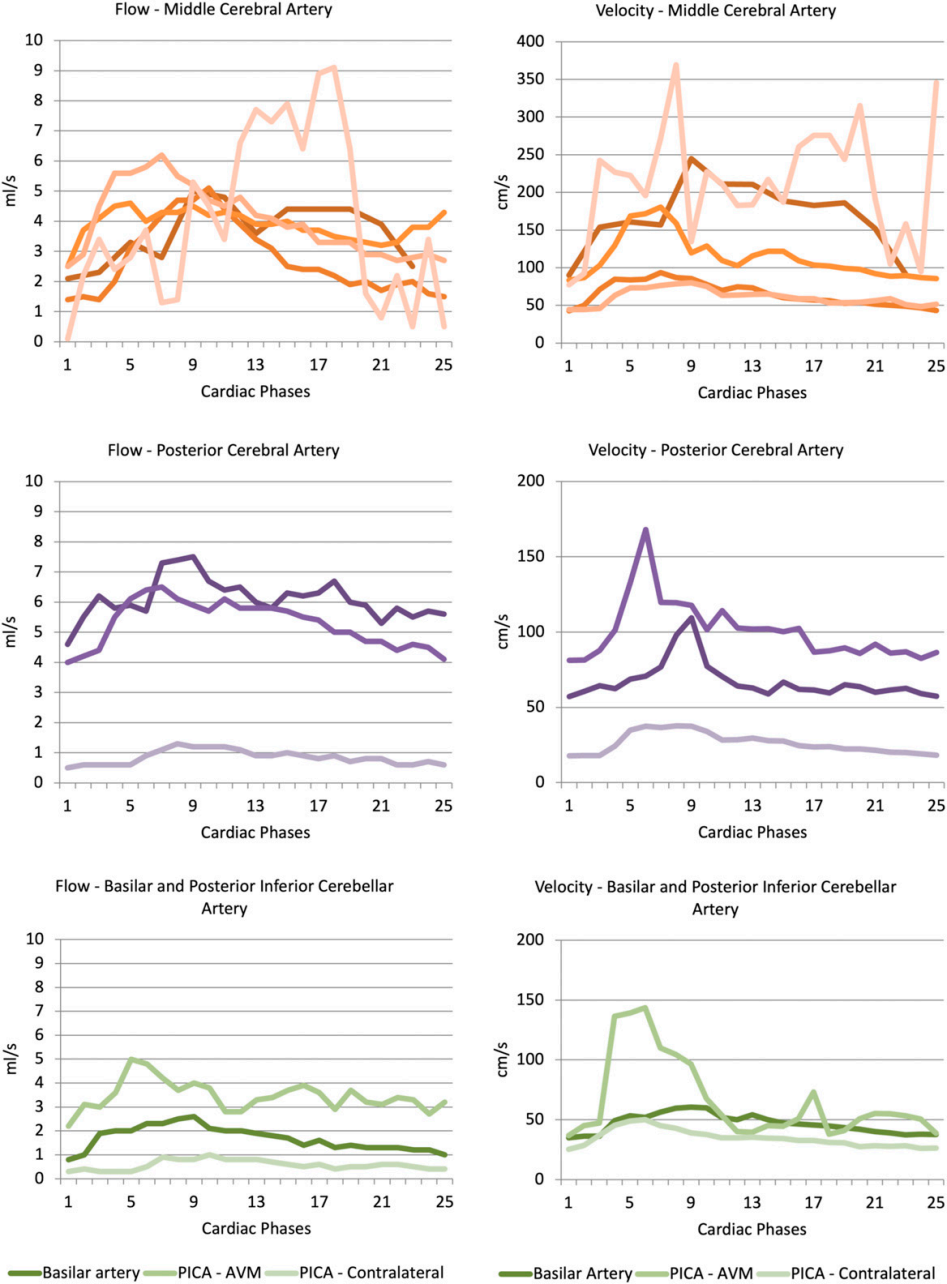
a–f. Curve representing flow and maximal velocity calculated for each of the 12 or 25 phases during the cardiac cycle in 10 patients. Graphs are separated according to feeding artery (middle cerebral artery, posterior cerebral artery, and basilar artery or posterior inferior cerebellar artery). Different shades of color represent different patients, except for PICA, which was measured on both the AVM side and contralateral side in the same patient.

Example MRI from one patient is shown in Figure 1. Results from the first MRI in all 10 patients are shown in Table 2 and Figure 2(a)–(f). The main feeding artery was the middle cerebral artery in five patients, posterior cerebral artery in three patients, and basilar artery and posterior inferior cerebellar artery in one patient. Results comparing patients from this study with healthy controls are shown in Table 3.

**Table 2.** Flow and velocity parameters measured in main feeding artery in patients with intracranial AVMs.

Main Feeding Artery	Highest Flow (mL/s)	Lowest Flow (mL/s)	Mean Flow (mL/s)	Peak Systolic Velocity (cm/s)	End-diastolic Velocity (cm/s)	Mean Velocity (cm/s)	Resistivity Index	Pulsatility Index
MCA	5.0	2.1	3.6	245	90	169	0.63	0.92
MCA	5.1	1.4	2.7	94	43	65	0.54	0.78
MCA	4.6	2.5	3.9	180	84	114	0.54	0.85
MCA	6.2	2.5	4.0	80	45	61	0.44	0.58
MCA	9.1	0.1	4.0	369	77	212	0.79	1.38
Mean all MCA	6.0	1.7	3.7	194	68	124	0.59	0.90
BA	2.6	0.8	1.7	61	35	47	0.42	0.55
PCA	7.5	4.6	6.1	109	57	67	0.48	0.78
PCA	6.5	4.0	5.3	168	81	101	0.52	0.86
PCA	1.3	0.5	0.8	38	18	26	0.53	0.77
Mean all PCA	5.1	3.0	4.1	105	52	65	0.51	0.80
PICA	5.0	2.2	3.5	144	37	66	0.74	1.61

MCA - middle cerebral artery, BA - basilar artery, PCA - posterior cerebral artery, PICA - posterior inferior cerebellar artery.

**Table 3.** Results in patients with AVMs in this study compared with healthy controls.

Main Feeding Artery	Nidus Volume (cm^3^)	Percent of healthy controls with higher flow (%)			Percent of healthy controls with higher velocity (%)			Percent of healthy controls with higher index (%)	
		Highest Flow	Lowest Flow	Mean Flow	Peak Systolic Velocity	End- diastolic Velocity	Mean Velocity	Resistivity Index	Pulsatility Index
PCA	0.5	83	92	91	82	96	93	6	6
MCA	0.9	7	93	58	31	58	35	18	23
MCA	2.5	0	10	3	62	48	46	68	73
PCA	5.7	0	0	0	0	0	0	8	2
MCA	8.9	20	10	4	0	0	0	20	16
MCA	9.3	9	34	7	0	0	0	6	12
PCA	29.6	0	0	0	0	0	0	22	5
MCA	92.9	0	100	3	0	0	0	0	2

Patients are ordered according to nidus volume. PCA - posterior cerebral artery, MCA - middle cerebral artery.

There were statistically significant correlations between nidus volume and flow and velocity parameters (Spearman correlation coefficient 0.64 for maximal flow, 0.73 for mean flow, 0.76 for PSV, 0.77 for EDV, and 0.83 for mean velocity). No significant correlation was found between nidus volume and lowest flow, RI, or PI.

**Figure 3.**
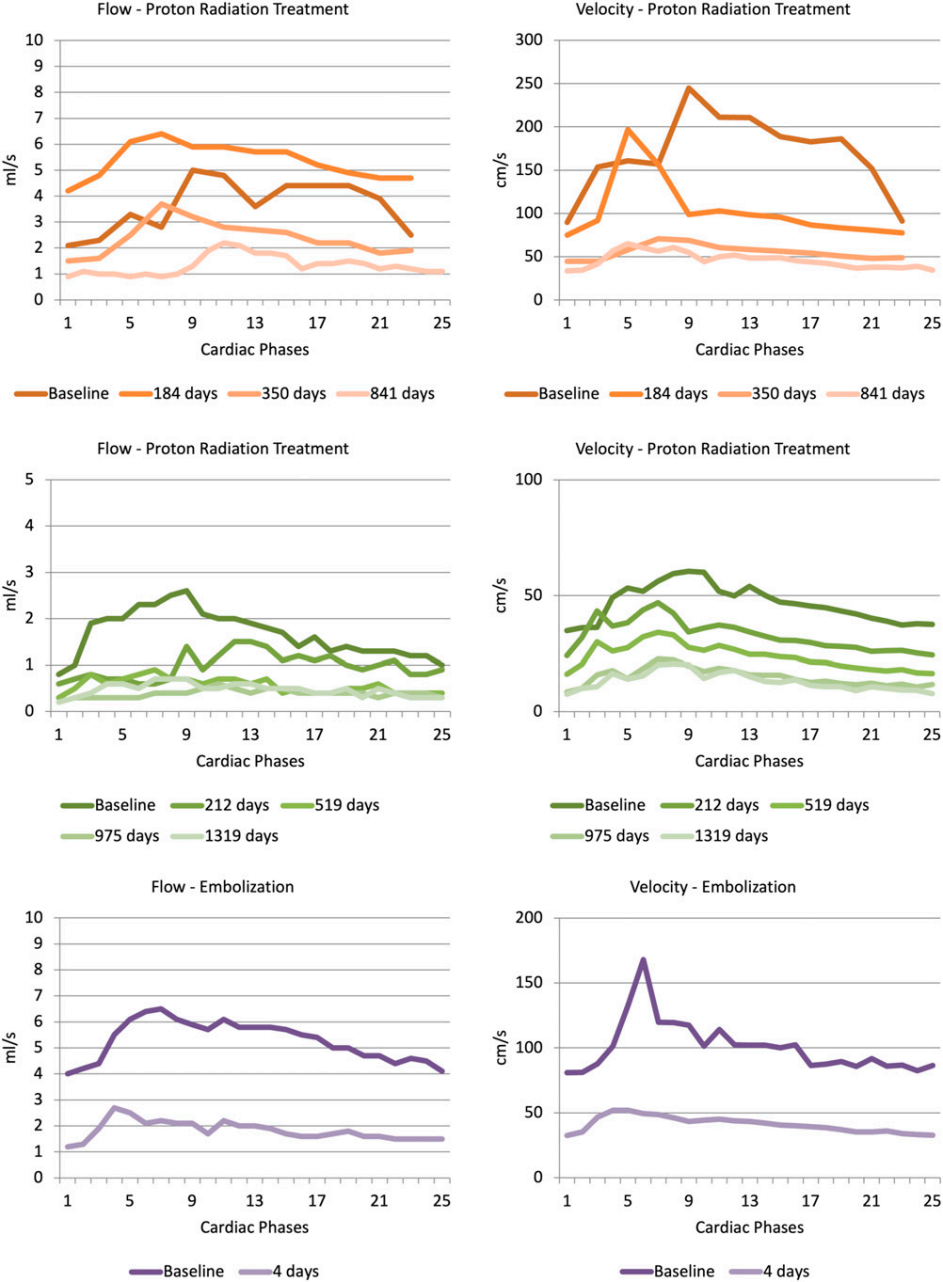
a–f. Curve representing flow and maximal velocity calculated for each of the 12 or 25 phases during the cardiac cycle in three different patients. Patients received treatment for their AVMs, and phase-contrast MRI was done at baseline and follow-up at different timepoints after treatment.

**Figure 4.**
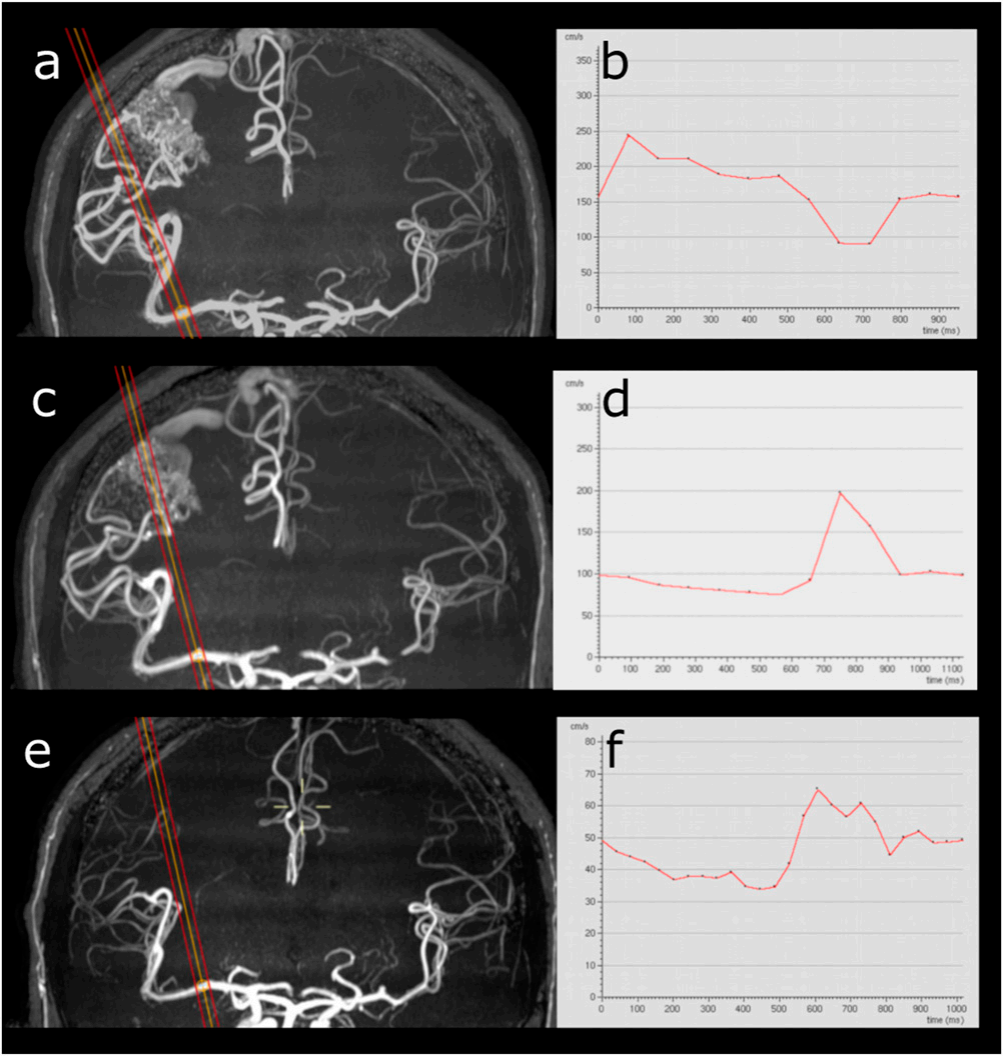
a–f. Subject with an AVM with the middle cerebral artery as the feeding vessel at baseline (a–b), with follow-up at 6 months (c–d) and 12 months (e–f) after treatment with proton radiation. Left column—maximum intensity projections of 3D time-of-flight MR angiography sequence showing the AVM nidus in the left parietal lobe, with orange and red lines illustrating the placement of the measuring plane. Right column—curves representing maximal velocity over the cardiac cycle obtained at each corresponding time point.

Three patients were examined both before and after treatment. Two patients received treatment with proton radiation, and one patient was treated with partial embolization of the nidus. Results in these three patients are shown in Table 4 and Figure 3(a)–(f). Example pre- and post-treatment MRI from one patient is shown in Figure 4. Table 5 shows the results of comparing measurements after treatment in patients in this study with healthy controls.

**Table 4.** Flow and velocity parameters measured in main feeding artery before and after treatment in patients with intracranial AVMs.

Main Feeding Artery	Type of treatment	Days after treatment	Highest Flow (mL/s)	Lowest Flow (mL/s)	Mean Flow (mL/s)	Peak Systolic Velocity (cm/s)	End- diastolic Velocity (cm/s)	Mean Velocity (cm/s)	Resistivity Index	Pulsatility Index
MCA	Proton radiation		5.0	2.1	3.6	245	90	169	0.63	0.92
		184	6.4	4.2	5.4	197	75	104	0.62	1.18
		350	3.7	1.5	2.4	71	45	55	0.37	0.47
		841	2.2	0.9	1.3	65	34	46	0.48	0.68
BA	Proton radiation		2.6	0.8	1.7	61	35	47	0.42	0.55
		212	1.5	0.6	1.0	47	24	33	0.49	0.69
		519	0.9	0.3	0.6	34	16	24	0.53	0.76
		975	0.6	0.2	0.4	23	9	15	0.63	0.96
		1319	0.7	0.2	0.5	21	8	13	0.63	0.99
PCA	Embolization		6.5	4.0	5.3	168	81	101	0.52	0.86
		4	2.7	1.2	1.8	52	33	41	0.37	0.47

MCA - middle cerebral artery, BA - basilar artery, PCA - posterior cerebral artery.

**Table 5.** Results in two treated patients with AVMs in this study compared with healthy controls.

Main Feeding Artery	Type of treatment	Days after treatment	Nidus Volume (cm^3^)	Percent of healthy controls with higher flow (%)			Percent of healthy controls with higher velocity (%)			Percent of healthy controls with higher index (%)	
				Highest Flow	Lowest Flow	Mean Flow	Peak Systolic Velocity	End-diastolic Velocity	Mean Velocity	Resistivity Index	Pulsatility Index
MCA	Proton radiation		9.3	9	34	9	0	0	0	6	12
MCA		184		0	0	0	0	0	0	8	5
MCA		350		60	89	78	85	47	68	94	92
MCA		841		99	99	100	93	93	95	45	45
PCA	Embolization		5.7	0	0	0	0	0	0	8	1
PCA		4		2	8	5	36	19	29	81	76

MCA - middle cerebral artery, PCA - posterior cerebral artery.

## Discussion

In this study, we measured flow and velocity with cardiac-gated PC-MRI in feeding arteries of AVMs. Large AVMs had higher flow and velocity, and velocity was superior to flow in discriminating between normal and pathological measurements. We found a decrease in both flow and velocity measurements after treatment. One of the strengths in our study is that we investigated several different flow parameters to better understand which parameter to use when trying to identify pathological values and monitor treatment response.

In our study, the mean flow was 5.8 mL/s in the middle cerebral artery and 4.1 mL/s in the posterior cerebral artery, values which are in accordance with previously published values. Increased mean flow, mean velocity, and peak velocity in feeding arteries have been observed in previous studies.^10,12–14^ Two studies reported comparable measurements and found flow in the middle cerebral artery to be 5.1 to 7.6 mL/s and 4.7 mL/s in the posterior cerebral artery.^10,12^ Variation in nidus size among studies can explain these small differences. In these studies, the authors also report mean velocity, but methodological differences make comparisons difficult because they report mean velocity as the mean values over the vessel lumen rather than the mean maximum voxel value.^10,12^ We chose to report the maximum value, as the values closest to the vessel wall were encumbered by artefacts in several cases, and in laminar flow, the maximum values are at the vessel center. These artefacts might be caused by turbulent flow adjacent to small branching vessels, vessel wall irregularities, or proximity to vessel division.

Significant correlations between nidus volume and flow and velocity parameters were seen in our study. Previous studies have also found side differences in mean flow in the internal carotid arteries in patients with medium or large AVMs (> 5 mL) and increasing mean flow and mean velocity with increasing nidus size.^19,20^ To our knowledge, no previous study has investigated the relation between nidus size and measurements in single intracranial arteries.

We found mean flow measurements of 1.7 to 6.0 mL/s and velocities of 52 to 194 cm/s in the middle and posterior cerebral arteries. Previous studies measuring flow and velocity with PC-MRI in anterior, middle, and posterior cerebral arteries in healthy subjects and post-stroke patients found lower mean values for flow and velocity compared to our study in patients with AVMs. There is, however, an overlap, and the highest measured flow and velocity values in the non-AVM population were higher than the lowest measured parameters in AVM patients.^7,8^ However, velocity measurements in patients with a large nidus differ from those seen in healthy subjects. We therefore believe that the measurement of velocity parameters helps to improve the discrimination between healthy and pathological values and the assessment of treatment effects. As mentioned above, the velocity measurements are also less likely to be affected by artefacts encountered at the vessel border.

In all three patients examined both before and after treatment, there was a decrease in flow and velocity measurements after treatment. In patients that receive radiation treatment for AVMs, there is a latency period before treatment effect. We could see a decrease in velocity parameters from the first follow-up examination and up to 2.7 years after treatment. In one patient, the first follow-up examination revealed an increase in flow, but in this examination ROI adjustments were made to exclude inverted intraluminal signal at the vessel border, and the remaining vessel area was smaller than on the previous examination. Previous studies have shown a decrease in flow and velocity parameters, but most studies have measured the internal carotid artery and not intracranial feeding arteries.^11,13,19–22^

We used cardiac-gated PC-MRI and were thus able to detect the fluctuations in flow and velocity over the cardiac cycle. We could therefore calculate not only mean flow and velocity but also the maximal and minimal values. Comparing the results from this study to previously published measurements in healthy individuals, we found velocity parameters to be more sensitive in distinguishing pathological from normal values. We believe that one reason for this result is that velocity measurements are not as sensitive to artifacts that require adjusting the ROI size. Previous studies using TCD have shown a reduced RI and PI in patients with AVMs.^2,23–26^ In this study, we found RI and PI in the lower range compared to healthy subjects, but we were not able to differentiate between healthy subjects and AVM patients. This might be due to the small patient population. No correlation between nidus size and RI and PI was found, suggesting that these values might be associated with other morphological aspects of the AVMs (e.g., venous drainage) or that these measurements in the proximal feeders do not reflect the measurements that would be obtained by measuring in the distal feeders closer to the AVM nidus.

PC-MRI can be performed with a 2D sequence as in this study or with a 3D sequence. Furthermore, it is possible to acquire a 3D PC sequence dynamically, that is, 4D. However, 4D PC-MRI has a longer acquisition time and requires advanced data processing and visualization tools. It is also possible to acquire time-averaged PC-MRI (non-gated images) both in a single imaging plane or as a volume. Time-averaged PC-MRI has the advantaged of a shorter acquisition time, but information regarding flow and velocity variation over the cardiac cycle cannot be obtained. In this study, we chose to use a commercially available 2D PC-MRI sequence that had an acquisition time that was applicable in a clinical setting. We used cardiac gating as we wanted to assess what parameter over the cardiac cycle was most useful in distinguishing patients from healthy controls.

Patients with AVMs have an increased risk for intracranial hemorrhage, with an annual bleeding risk of 2–3%.^27,28^ However, there are risks with treatment that may overweigh the benefits, as illustrated by the ARUBA trial.^29,30^ There is also a small but not negligible risk of rebleeding from treated AVMs with residual nidus.^31^ In patients with AVMs partially treated with embolization, there is a clinical difficulty in assessing the remaining nidus.^32,33^ With use of PC-MRI measurements in the feeding artery, it may be possible to obtain functional hemodynamic information of the AVM related to the pressure conditions inside the nidus.^34^ As the intranidal pressure has an important impact on the risk of rupture, hemodynamic information in the feeding artery may assist in the estimation of a patient’s individual risk of intracranial hemorrhage and help assess which patients would benefit from interventional therapy.

The main limitation is the small and heterogeneous patient population, which underlines the relatively rare pathology of intracranial AVMs. Due to limitations in scan time, we performed only unilateral measurements in most patients. ROI size adjustments, excluding part of the vessel lumen, were necessary in six MRI, and flow measurements were therefore affected in these patients. There was an evolution of scan technique during the study. A lower temporal resolution (12 cardiac phases instead of 25) was used in the healthy controls and the initial three MRI scans in this study (all in the same patient), which may have caused some underestimation of flow and velocity measurements. Another limitation of the study was the utilization of 2D flow imaging instead of 4D flow imaging, which offers a three-dimensional representation and the ability to perform multiple measurements within the acquired volume.

In conclusion, patients with a large AVM nidus have increased velocity measurements compared to healthy individuals. There is a reduction in both flow and velocity in feeding arteries after treatment. We believe that measurement of velocity parameters improves the discrimination between healthy and pathological values in patients with AVMs. Future studies in larger patient cohorts are needed to evaluate the usefulness of PC-MRI-measured velocity as a tool for risk stratification and evaluation of treatment response.

## References

[1] Nico E Hossa J McGuire LS , et al. Rupture-risk stratifying patients with cerebral arteriovenous malformations using quantitative hemodynamic flow measurements. World Neurosurg 2023; 179: 68–76. DOI: 10.1016/j.wneu.2023.08.047. 37597662

[2] Kaspera W Ladzinski P Larysz P , et al. Transcranial color-coded Doppler assessment of cerebral arteriovenous malformation hemodynamics in patients treated surgically or with staged embolization. Clin Neurol Neurosurg 2014; 116: 46–53. DOI: 10.1016/j.clineuro.2013.11.001. 24309152

[3] Dempsey RJ Moftakhar R Pozniak M . Intraoperative Doppler to measure cerebrovascular resistance as a guide to complete resection of arteriovenous malformations. Neurosurgery 2004; 55: 155–160. 15214984 10.1227/01.neu.0000126879.95006.46

[4] Carr HY Purcell EM . Effects of diffusion on free precession in nuclear magnetic resonance experiments. Phys Rev 1954; 94: 630–638.

[5] Nayler GL Firmin DN Longmore DB . Blood flow imaging by cine magnetic resonance. J Comput Assist Tomogr 1986; 10: 715–722. 3528245 10.1097/00004728-198609000-00001

[6] Zhao M Amin-Hanjani S Ruland S , et al. Regional cerebral blood flow using quantitative MR angiography. AJNR Am J Neuroradiol 2007; 28: 1470–1473. DOI: 10.3174/ajnr.A0582. 17846193 PMC8134363

[7] Correia de Verdier M Wikstrom J . Normal ranges and test-retest reproducibility of flow and velocity parameters in intracranial arteries measured with phase-contrast magnetic resonance imaging. Neuroradiology 2016; 58: 521–531. DOI: 10.1007/s00234-016-1661-6. 26882908

[8] Chang KH Lee YH Chen CY et al. Inter- and intra-rater reliability of individual cerebral blood flow measured by quantitative vessel-flow phase-contrast MRI. J Clin Med. 2020; 9(10 0): 3099. DOI: 10.3390/jcm9103099 32992892 PMC7601288

[9] Ansari SA Schnell S Carroll T , et al. Intracranial 4D flow MRI: toward individualized assessment of arteriovenous malformation hemodynamics and treatment-induced changes. AJNR Am J Neuroradiol 2013; 34: 1922–1928. DOI: 10.3174/ajnr.A3537. 23639564 PMC7965432

[10] Chang W Loecher MW Wu Y , et al. Hemodynamic changes in patients with arteriovenous malformations assessed using high-resolution 3D radial phase-contrast MR angiography. AJNR Am J Neuroradiol 2012; 33: 1565–1572. DOI: 10.3174/ajnr.A3010. 22499844 PMC6278605

[11] Shakur SF Amin-Hanjani S Abouelleil M , et al. Changes in pulsatility and resistance indices of cerebral arteriovenous malformation feeder arteries after embolization and surgery. Neurol Res 2017; 39: 7–12. DOI: 10.1080/01616412.2016.1258970. 27866455

[12] Shakur SF Amin-Hanjani S Mostafa H , et al. Relationship of pulsatility and resistance indices to cerebral arteriovenous malformation angioarchitectural features and hemorrhage. J Clin Neurosci 2016; 33: 119–123. DOI: 10.1016/j.jocn.2016.02.034. 27595365

[13] Wasserman BA Lin W Tarr RW , et al. Cerebral arteriovenous malformations: flow quantitation by means of two-dimensional cardiac-gated phase-contrast MR imaging. Radiology 1995; 194: 681–686. DOI: 10.1148/radiology.194.3.7862962. 7862962

[14] Wu C Ansari SA Honarmand AR , et al. Evaluation of 4D vascular flow and tissue perfusion in cerebral arteriovenous malformations: influence of Spetzler-Martin grade, clinical presentation, and AVM risk factors. AJNR Am J Neuroradiol 2015; 36: 1142–1149. DOI: 10.3174/ajnr.A4259. 25721076 PMC8013038

[15] Gosling RG King DH . Arterial assessment by Doppler-shift ultrasound. Proc R Soc Med 1974; 67: 447–449. 4850636 10.1177/00359157740676P113 PMC1645777

[16] Pourcelot L. Applications cliniques de l’examen Doppler transcutané. Vélocimétrie ultrasonore Doppler. Paris: Editions INSERM, 1975, pp. 213–240.

[17] Benaglia T Chauveau D Hunter DR , et al. Mixtools: An*R*Package for analyzing finite mixture models. J Stat Softw 2009; 32: 1–29. DOI: 10.18637/jss.v032.i06.

[18] R Core Team . R: a language and environment for statistical computing. Vienna, Austria: R Foundation for Statistical Computing, 2021.

[19] Schuster L Schenk E Giesel F , et al. Changes in AVM angio-architecture and hemodynamics after stereotactic radiosurgery assessed by dynamic MRA and phase contrast flow assessments: a prospective follow-up study. Eur Radiol 2011; 21: 1267–1276. DOI: 10.1007/s00330-010-2031-0. 21181407

[20] Marks MP Pelc NJ Ross MR , et al. Determination of cerebral blood flow with a phase-contrast cine MR imaging technique: evaluation of normal subjects and patients with arteriovenous malformations. Radiology 1992; 182: 467–476. DOI: 10.1148/radiology.182.2.1732966. 1732966

[21] Li CQ Hsiao A Hattangadi-Gluth J , et al. Early hemodynamic response assessment of stereotactic radiosurgery for a cerebral arteriovenous malformation using 4D flow MRI. AJNR Am J Neuroradiol 2018; 39: 678–681. DOI: 10.3174/ajnr.A5535. 29371257 PMC7410784

[22] Alaraj A Amin-Hanjani S Shakur SF , et al. Quantitative assessment of changes in cerebral arteriovenous malformation hemodynamics after embolization. Stroke 2015; 46: 942–947. DOI: 10.1161/strokeaha.114.008569. 25744522

[23] Petty GW Massaro AR Tatemichi TK , et al. Transcranial Doppler ultrasonographic changes after treatment for arteriovenous malformations. Stroke 1990; 21: 260–266. 2406994 10.1161/01.str.21.2.260

[24] Mast H Mohr JP Thompson JL , et al. Transcranial Doppler ultrasonography in cerebral arteriovenous malformations. Diagnostic sensitivity and association of flow velocity with spontaneous hemorrhage and focal neurological deficit. Stroke 1995; 26: 1024–1027. DOI: 10.1161/01.str.26.6.1024. 7762018

[25] Uggowitzer MM Kugler C Riccabona M , et al. Cerebral arteriovenous malformations: diagnostic value of echo-enhanced transcranial Doppler sonography compared with angiography. AJNR Am J Neuroradiol 1999; 20: 101–106. 9974063

[26] Jo KI Kim JS Hong SC , et al. Hemodynamic changes in arteriovenous malformations after radiosurgery: transcranial Doppler evaluation. World Neurosurg 2012; 77: 316–321. DOI: 10.1016/j.wneu.2011.06.061. 22120337

[27] Gross BA Du R . Natural history of cerebral arteriovenous malformations: a meta-analysis. J Neurosurg 2013; 118: 437–443. DOI: 10.3171/2012.10.jns121280. 23198804

[28] Kim H Al-Shahi Salman R McCulloch CE , et al. Untreated brain arteriovenous malformation: patient-level meta-analysis of hemorrhage predictors. Neurology 2014; 83: 590–597. DOI: 10.1212/wnl.0000000000000688. 25015366 PMC4141996

[29] Mohr JP Overbey JR Hartmann A , et al. Medical management with interventional therapy versus medical management alone for unruptured brain arteriovenous malformations (ARUBA): final follow-up of a multicentre, non-blinded, randomised controlled trial. Lancet Neurol 2020; 19: 573–581. DOI: 10.1016/s1474-4422(20)30181-2. 32562682

[30] Mohr JP Parides MK Stapf C , et al. Medical management with or without interventional therapy for unruptured brain arteriovenous malformations (ARUBA): a multicentre, non-blinded, randomised trial. Lancet 2014; 383: 614–621. DOI: 10.1016/s0140-6736(13)62302-8. 24268105 PMC4119885

[31] Choi SK Lim YJ Koh JS , et al. Post-treatment bleeding of cerebral arteriovenous malformations after Gamma Knife radiosurgery. J Korean Neurosurg Soc 2004; 36: 363–368.

[32] De Leacy R Ansari SA Schirmer CM , et al. Endovascular treatment in the multimodality management of brain arteriovenous malformations: report of the society of neurointerventional surgery standards and guidelines committee. J Neurointerv Surg 2022; 14: 1118–1124. DOI: 10.1136/neurintsurg-2021-018632. 35414599

[33] Shtraus N Schifter D Corn BW , et al. Radiosurgical treatment planning of AVM following embolization with Onyx: possible dosage error in treatment planning can be averted. J Neuro Oncol 2010; 98: 271–276. DOI: 10.1007/s11060-010-0177-x. 20383557

[34] Lv X Wu Z Li Y . Arteriovenous malformation in the brain: a theoretical study explaining the behavior of liquid embolic agents during endovascular treatment. NeuroRadiol J 2013; 26: 661–668. DOI: 10.1177/197140091302600609. 24355185 PMC4202870

